# Paradoxical Roles of the MAL/Tirap Adaptor in Pathologies

**DOI:** 10.3389/fimmu.2020.569127

**Published:** 2020-09-25

**Authors:** Imène Belhaouane, Eik Hoffmann, Mathias Chamaillard, Priscille Brodin, Arnaud Machelart

**Affiliations:** ^1^Univ. Lille, CNRS, INSERM, CHU Lille, Institut Pasteur de Lille, U1019 - UMR 9017 - CIIL - Center for Infection and Immunity of Lille, Lille, France; ^2^Laboratory of Cell Physiology, INSERM U1003, University of Lille, Lille, France

**Keywords:** TLRs, MAL/Tirap, chronic diseases, signaling pathways, inflammation

## Abstract

Toll-like receptors (TLRs) are at the forefront of pathogen recognition ensuring host fitness and eliciting protective cellular and humoral responses. Signaling pathways downstream of TLRs are tightly regulated for preventing collateral damage and loss of tolerance toward commensals. To trigger effective intracellular signaling, these receptors require the involvement of adaptor proteins. Among these, Toll/Interleukin-1 receptor domain containing adaptor protein (Tirap or MAL) plays an important role in establishing immune responses. Loss of function of MAL was associated with either disease susceptibility or resistance. These opposite effects reveal paradoxical functions of MAL and their importance in containing infectious or non-infectious diseases. In this review, we summarize the current knowledge on the signaling pathways involving MAL in different pathologies and their impact on inducing protective or non-protective responses.

## Introduction

Commensal and pathogenic microorganisms contain pathogen-associated molecular patterns (PAMPs) that are recognized by different families of pattern-recognition receptors (PRRs) (1). Mammals have distinct classes of PRRs, including Toll-like receptors (TLRs), that are patrolling both, extracellular and intracellular environments. They are expressed in innate immune cells, such as dendritic cells (DCs) and macrophages, but also in non-immune cells, such as fibroblasts and epithelial cells. TLRs are composed of the Toll/Interleukin-1 receptor homology (TIR) domain capable of binding to intracellular signaling adaptor proteins. For more details on downstream signaling cascades, we direct the reader to comprehensive reviews (2, 3).

**Figure 1:**
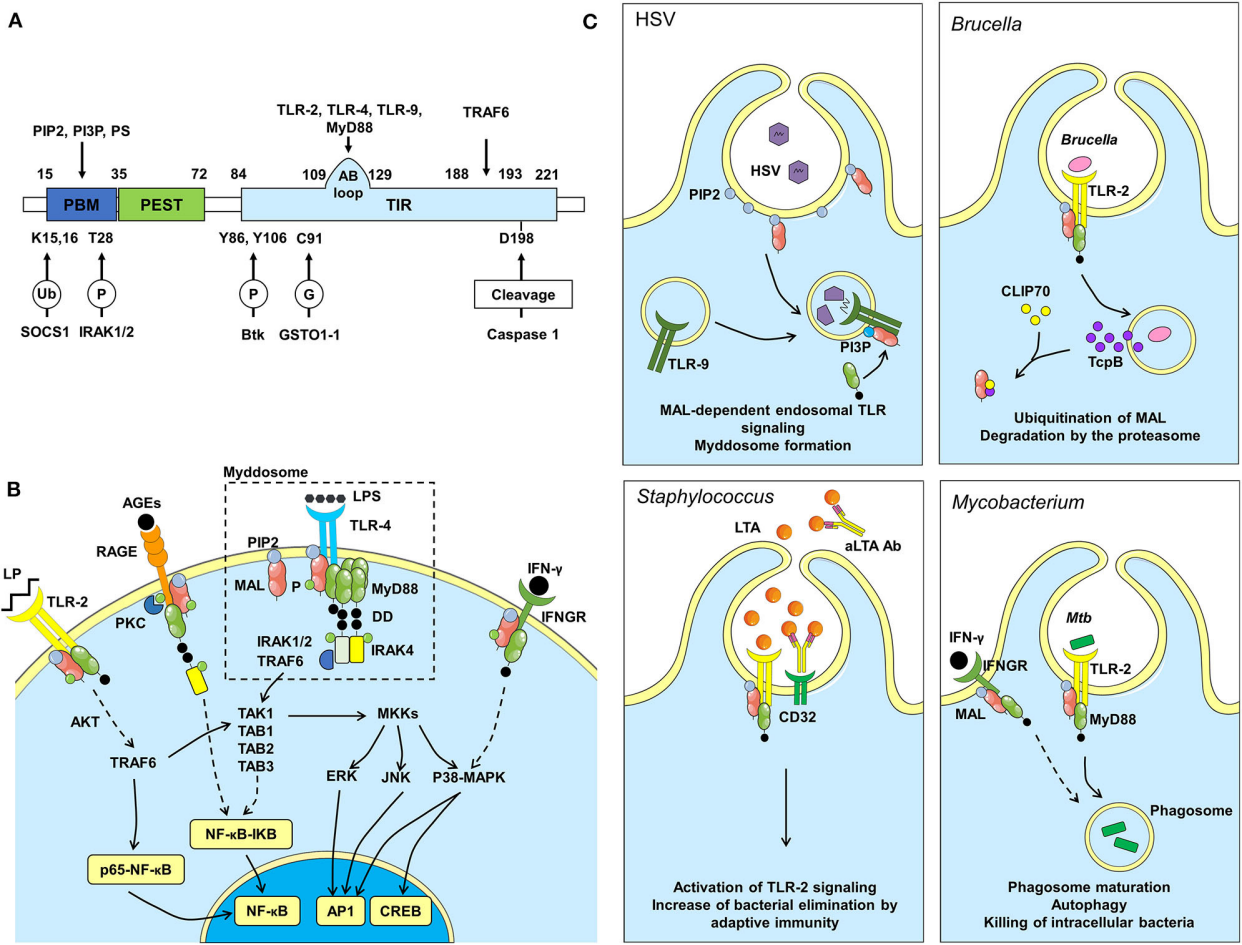
MAL structure, pathway and dependent immune responses during infections. **(A)** Protein structure of MAL, binding sites (above) and regulation sites (below). P, phosphorylation; Ub, ubiquitination; G, S-glutathionylation. **(B)** Among other receptors, MAL affects signals downstream of TLR-2, TLR-4, RAGE and IFNGR. When TLR-4 is activated by LPS, MAL is recruited by its PIP2 binding domain to TLR-4 rich regions of the plasma membrane. MAL then facilitates the recruitment of MyD88 and the formation of the myddosome, which is important for the activation of the NF-κB pathway, thus inducing inflammation. Signaling pathway activated downstream of RAGE can also induce NF-κB dependent inflammation. MAL induces the translocation of p65-NF-κB to the nucleus through AKT phosphorylation, independently from MyD88 signaling. Also, independently from MyD88, MAL can activate CREB via p38-MAPK and Mitogen-activated protein kinase kinase (MKK) signaling pathways. Another pathway involving MAL is downstream IFNGR, which leads to phosphorylation of p38-MAPK. AGEs, Advanced Glycation End products; DD, Death Domain; P, Phosphate group; LP, Lipopeptide. **(C)** The *Herpes simplex* virus (HSV) infection model was used to confirm MAL signaling from endosomes. TLRs found at the cell surface signal from a PIP2-rich subdomain, and MAL is recruited to that location via interactions with PIP2. TLRs found on endosomes (TLR-9) signal from a domain rich in PI3P. These lipids direct MAL to endosomes to promote TLR-9 signaling after viral DNA recognition. *Brucella* infection: *Brucella* interferes with immune responses by producing TcpB, which targets MAL to inhibit NF-κB activation. TcpB also targets CLIP70 inducing MAL ubiquitination and degradation by the proteasome. *Staphylococcus* infection: Lipoteichoic acid (LTA), a toxin produced by *Staphylococcus* bacteria, is recognized by TLR-2. TLR-2 activation induces MAL signaling to eliminate the toxin/bacteria. LTA is also recognized by antibodies (aLTA Ab), which are recognized by CD32 (Fcγ receptor II). In patients carrying the R121W mutation in MAL, adaptive immunity can compensate for defects in MAL function. *Mycobacterium* infection: Killing *M. tuberculosis* requires activation of TLR-2- and IFNGR-dependent signaling pathways within phagocytes to induce phagosome maturation and autophagy.

Among a variety of TLRs adaptor proteins, TIR-containing adaptor protein (Tirap), also named MyD88 adaptor-like protein [MAL, also referred to as megakaryoblastic leukemia (translocation) 1], was mostly reported for its involvement in the regulation of signaling cascades downstream of TLR-2 and TLR-4 by bridging the adaptor protein myeloid differentiation primary response 88 (MyD88) (4–6). Human MAL consists of 221 amino acids (Figure 1A). At the N-terminus, there are a phosphatidylinositol 4,5-bisphosphate (PIP2) binding motif (PBM) and a putative proline, glutamic acid, serine and threonine (PEST) domain associated to short-lived proteins (7). At the C-terminus, a TIR domain extends from amino acid 84–221 and orchestrates the signal transduction pathways after TLR and Interleukin-1 (IL-1) receptor engagement. Because of its analogy with MyD88, MAL signaling was initially confused with that of MyD88. Actually, it impacts on inflammation and innate immune responses in a TLR- and MyD88-independent manner (8), pinpointing its role to other putative cellular mechanisms, such as vesicular trafficking. Heterozygosity and homozygosity for some inherited mutations in *MAL* are associated with different outcomes in patients, suggesting a paradoxical role in protection against diseases. This may reflect the influence of gene-gene and gene-environment interactions that vary across populations. In this review, we summarize the current knowledge on cellular mechanisms of MAL/Tirap and highlight its role in disease predisposition.

## MAL Signaling Pathways Downstream TLR-2 and TLR-4

MAL recruitment at the plasma membrane occurs upon binding to PIP2 prior to its interaction with TLRs (Figure 1B) (9). MAL is then phosphorylated by Burton tyrosine kinase (Btk), which facilitates its interaction with the TIR domain of TLRs and consequently MyD88 to initiate the transduction signal (10). Recently, it has been shown that MAL is S-glutathionylated on Cys91 to facilitate the interaction with MyD88 (11). Notably, it is possible that the MAL-MyD88 tandem is prepositioned on the membrane awaiting ligand-induced TLR activation. Upon activation, MyD88 oligomerizes to form a large signaling platform called the “myddosome,” which also contains members of the Interleukin-1-Receptor-Associated Kinase (IRAK) family. The N-terminal Death Domain of MyD88 recruits firstly IRAK4 and then IRAK1 and IRAK2, which are phosphorylated to interact with the TNF receptor-associated factor 6 (TRAF6). TRAF6 is an E3 ubiquitin ligase able to generate K63-linked polyubiquitination chains. The linear ubiquitin assembly complex can bind these chains to recruit preassembled kinase complexes containing TAK1, TAB1, TAB2, and TAB3. This leads to nuclear factor kappa B (NF-κB) translocation to the nucleus after inhibitor of NF-κB (IKB) proteolytic degradation. Concomitantly, this complex controls the mitogen-activated protein kinase (MAPK) signaling that activates members of the activator protein-1 (AP1) transcription factor family, Jun and Fos, resulting in cytokine production, initiation of inflammation and metabolic cell polarization (6, 12).

Thus, MAL is an important actor in the establishment of inflammation. Over the last decade, a large number of clinical and experimental studies focused on the role of MAL in the control of infectious and non-infectious diseases (Table 1). Unexpectedly, as shown in Table 1, genetic variation in *MAL* was associated with either protection or susceptibility to diseases. Recent studies have shown that MAL is involved in other processes besides TLR-2 and TLR-4 making its biology more complex than expected. These new functions, discussed below, will make it possible to emit new hypotheses explaining the paradoxical behavior of MAL.

**Table 1:** Effect of MAL deficiency during infectious and non-infectious diseases.

**Host**	**Pathogen**	**Disease**	**Genotype**	**Effect**	**References**
Mice	*Escherichia coli*	Bacteraemia	MAL/Tirap Knock-out	Protection	(13)
	*Pseudomonas aeruginosa*	–	MAL/Tirap Knock-out	None	(14)
	*Klebsiella pneumoniae*	Pneumonia	MAL/Tirap Knock-out	Protection	(14)
	*Bordetella pertussis*	Whooping cough	MAL/Tirap Knock-out	Protection	(15)
	*Salmonella enterica*	Salmonellosis	MAL/Tirap Knock-out	None	(16, 17)
			MAL/Tirap Knock-out	Protection	(18)
	*Mycobacterium tuberculosis*	Tuberculosis	MAL/Tirap Knock-out	None	(19)
			MAL/Tirap S200L	Susceptibility	(8)
	*Tripanosoma cruzi*	Chagas disease	MAL/Tirap Knock-out	Protection	(20)
	*Herpes simplex*	Herpes	MAL/Tirap Knock-out	predicted susceptibility	(21)
	–	Colorectal cancer	MAL/Tirap Knock-out	Susceptibilty	(22)
Human	*Mycobacterium tuberculosis*	Tuberculosis	MAL/Tirap S180L	None	(23–27)
			MAL/Tirap S180L	Susceptibility	(28)
			MAL/Tirap S180L	Protection	(29–32)
			MAL/Tirap S55N	None	(23)
			MAL/Tirap D96N	Susceptibility	(26)
			MAL/Tirap A186A	Susceptibility	(23)
	*Streptococcus pneumoniae*	Pneumococcal disease	MAL/Tirap S180L	Protection	(24, 33)
			MAL/Tirap 180L homozygous	Susceptibility	(33)
	*Trypanosoma cruzi*	Chagas disease	MAL/Tirap S180L	Protection	(34)
	*Plasmodium falciparum*	Malaria	MAL/Tirap S180L	Protection	(24, 35, 36)
			MAL/Tirap S180L	None	(37)
			MAL/Tirap S180L	Susceptibility	(38, 39)
	*Haemophilus influenzae* B	Vaccine failure	MAL/Tirap S180L	Potection	(40, 41)
	*HIV-1*	AIDS	MAL/Tirap S180L	Protection	(42)
	*Staphylococcus aureus*	Staphylococcal disease	MAL/Tirap R121W	Susceptibility	(43)
	*Helicobacter pylory*	Gastritis and peptic ulcer	MAL/Tirap S180L	Protection	(44)
	–	Lupus Erythematosus	MAL/Tirap S180L	Protection	(25, 45)
	–	Rheumatoid arthritis	MAL/Tirap S180L	None	(40)
			MAL/Tirap overexpression	Susceptibility	(46)
	–	Behçet's disease	MAL/Tirap S180L	Protection	(47)
			MAL/Tirap S180L	None	(48)
	–	Gastric cancer	MAL/Tirap C558T	Susceptibility	(49)
	–	Lymphoma	MAL/Tirap R81C	Susceptibility	(50)
	–	Atopic dermatitis	MAL/Tirap S180L	Protection	(51)
			MAL/Tirap Q101Q	Protection	(51)

## Does MAL Signal Through Endosomal TLRs?

Several studies have investigated whether intracellular TLRs require MAL to signal. While PIP2 is required for MAL recruitment at TLR-2 and TLR-4 (10, 52), functional analysis by Kagan's group revealed that Phosphatidylinositol 3-phosphate (PI3P) and Phosphatidylserine (PS) are needed to recruit MAL to endosomal TLR-9 (21). The authors used MAL-deficient plasmacytoid DCs, known to respond exclusively via endosomal TLRs, to demonstrate that MAL is required for production of type I interferon (IFN) downstream TLR-9 stimulation with *Herpes simplex* Virus (HSV) (Figure 1C). Wild type and MAL-deficient primary bone marrow-derived macrophages were stimulated with substrains of HSV-1 showing that deficient cells presented a defective production of IL-1β and IL-6, specifically downstream of endocytic TLR-9 (21, 53). Since then, it became clear that multiple targets of the lipid-binding domain of MAL are functionally important and allow this adaptor to promote TLR signaling from both plasma membrane and endosomal compartments. Corroborating this, Shan et al., also demonstrated that MAL was recruited as an adaptor to endosomal compartments by TLR-8 (54). The complexity of the endosomal system fine-tunes the immune response by ensuring the proper compartmentalization of intracellular TLRs. The contribution of MAL downstream of intracellular TLRs increases the complexity of its biology and further investigations are needed to fully understand the regulation of endosomal signaling which could provide new hypotheses explaining the paradoxical role of MAL.

## Could MAL Signaling Be Independent of TLRs?

Besides the above-mentioned MAL-mediated pathways that are downstream of TLRs, Keane's group discovered in murine macrophages that MAL binds to IFN-γ receptor (IFNGR), which triggered its interaction with MyD88 (Figure 1B). In the context of *Mycobacterium tuberculosis* (*Mtb*) infection, authors reported that MAL leads to autophagy and vacuole acidification that kills the bacteria (Figure 1C). In addition, the S180L polymorphism (this single-nucleotide polymorphism (SNP) encodes a serine instead of a leucine), and its murine equivalent S200L, compromised IFNGR signaling impairing host responses to *Mtb* (8).

MAL was also described to interact with Receptor for Advanced Glycation End products (RAGE), a type I single-pass transmembrane protein belonging to the immunoglobulin superfamily (Figure 1B). Upon binding of the extracellular domain of RAGE to its ligands, the cytoplasmic domain of this receptor is phosphorylated at Ser391 by PKCζ. Consequently, this leads to the recruitment of MAL and MyD88, further inducing a recruitment of IRAK4, activation of the downstream effector kinases and finally production of inflammatory cytokines through activation of NF-κB. RAGE and TLR-2/4 partly share an intracellular signaling pathway. These receptors display a precise motif in the intracellular domain (Q residue followed by three successive negatively charged residues), which, upon phosphorylation, enhances its affinity to MAL (55, 56). During infection, such as tuberculosis (Tb), the RAGE pathway is modulated (57). Since MAL is implicated in this signaling pathway, mutations in the adaptor could certainly impact on disease severity. It was shown that PKCζ is upregulated during Tb progression, which increases effector killing functions (58). As PKCζ facilitates the recruitment of MAL to RAGE, it becomes an interesting way of investigation to decipher MAL's role during *Mtb* infection. Furthermore, co-morbidities such as diabetes are known to increase the amount of RAGE ligands, which could influence MAL functions during infection (59). Based on these observations, we can legitimately assume that MAL is implicated in other unknown pathways.

## How is MAL Regulated and Degraded?

The PEST domain of MAL undergoes phosphorylation and polyubiquitination of lysine residues targeting degradation *via* the 26S proteasome. Mansell et al., demonstrated that stimulation of both TLR-2 and TLR-4 induced MAL degradation within 15–30 min after stimulation in order to avoid chronic inflammation. The degradation of MAL is a consequence of its polyubiquitination, which occurs via the SH2 domain of SOCS-1 and subsequent recruitment of the ubiquitin machinery (60). Tyrosine phosphorylation of MAL *via* Btk is necessary for the SOCS-1–mediated degradation. Moreover, MAL ubiquitination and degradation was also shown to be mediated by Cytoplasmic Linker Protein 170 (CLIP170) that is implicated in regulation of microtubule dynamics, cell migration and intracellular transport (7, 61). A study also demonstrated that MAL phosphorylation at Thr28 within its PBM reduces PI interactions and cell membrane targeting, leading to its ubiquitination and degradation (62).

Moreover, limiting the amount of PIP2 at the plasma membrane would prevent an exacerbated inflammation. Aksoy et al., showed in DCs that the interaction of MAL with phosphoinositol-3 kinases (PI3K) converts PIP2 to PIP3 and leads to the shedding of its membrane anchor sites. The change in the PIP2/PIP3 ratio favored the redistribution of MAL into the cytosol, where it was thus degraded. Moreover, TLR-4 is internalized, inactivating its downstream signaling pathway (1, 7).

## Are TLR/MAL-Derived Inflammatory Responses Helpful in Fighting Diseases?

In the context of whooping cough, a pulmonary infection caused by *Bordetella pertussis*, the absence of MAL induced susceptibility to the infection in mouse lungs with exacerbated dissemination leading to death (15). In this setting, MAL impacted on early local pro-inflammatory cytokine production by alveolar macrophages in lungs and prevented apoptosis-induced cell death and depletion of alveolar macrophages. As expected through this example, loss of function in MAL decreases the inflammatory response which leads to susceptibility to the infection. However, MAL biology is not as straightforward in other models for which the level of TLR/MAL-derived inflammation will strongly impact the severity of the disease. The impact of S180L polymorphism was deeply investigated. In a group of Pakistani population, it was observed that the 180L allele frequency is higher than that of the 180S allele in patients infected with *Plasmodium*, the causative agent of malaria, demonstrating that MAL deficiency enhances 3.000 times the chance of acquiring malaria caused by *Plasmodium falciparum* (35). In a cohort of adults from India, the heterozygous S180L mutation led to an optimal release of TNF-α that was shown to be protective against severe *P. falciparum* infection and mortality (36). Several studies also focused on the implication of MAL S180L polymorphism during Tb resulting in confusing conclusions. Indeed, S180L SNP has been often associated with protection. Capparelli et al., demonstrated that MAL S180L conferred resistance against Tb in heterozygous individuals, showing that those subjects displayed intermediate levels of IFN-γ, TNF-α or nitric oxide (NO), which helped to control the infection (29). Together, these results suggest that an intermediate level of inflammation decreases the severity of the pathology leading to a better protection to some pathogens that normally take advantage of an exacerbated inflammation.

Whereas MAL signaling was long considered important for antimicrobial immunity, recent studies demonstrated that genetic impairments in *MAL* are also associated with tumorigenesis. Interestingly, MAL overexpression was reported in ~20% of investigated lymphoma, and a whole exome sequencing in human recently revealed that MAL SNP R81C activated its downstream signaling to enhance NF-κB gene expression, whose constitutive activity is characteristic of B cell lymphoma (50, 63). It became clear that TLR/MAL-dependent inflammation strongly influences disease control with versatile consequences.

## Does MAL Have Immunoregulatory Properties?

Following the hypothesis that negative feedback could avoid chronic inflammation and septic shock, a putative immunoregulatory role of MAL was investigated (20, 64). Mellett et al., demonstrated that MAL is the unique TIR adaptor protein capable of activating cyclic adenosine monophosphate (c-AMP) Response Element-binding proteins (CREB), a key transcription factor that mediates regulation of gene expression. They showed that MAL-induced phosphorylation of CREB was induced by LPS that stimulated TLR-4, suggesting a positive feedback system in dysregulated inflammatory responses, where MAL induces the production of IL-10 and cyclooxygenase 2 (COX-2). During *Trypanosoma cruzi* infection, the causative agent of Chagas disease, it was shown in a mouse model of infection that MAL deficiency is associated to exacerbated inflammation, similarly to TLR-2 deficient mice, leading to decreased parasitemia and delayed mortality (20). Moreover, authors distinguished between pro-inflammatory LyC6^hi^TLR2^hi^ and anti-inflammatory Ly6C^lo^TLR2^hi^ splenic monocytes and demonstrated that MAL was associated with cytokine production by the immunosuppressive population after triggering TLR-2 or TLR-9.

The confusing role of MAL could be explained by the fact that MAL induces both pro-inflammatory and anti-inflammatory responses depending on the stimulated receptor and the targeted cell population. For example, *Mtb* is known to colonize different cell types and organs depending on the chronicity of the infection (65), possibly inducing different levels of inflammation. For instance, Russell's team demonstrated that *Mtb* grows differentially within interstitial macrophages compared to alveolar macrophages, which are more permissive to infection (66). The involvement of MAL in the immune response to *Mtb* infection in these two macrophage subtypes could provide new insights in the versatility of MAL functions.

## Can Adaptive Immune Responses Compensate a Lack of Innate Immune Responses?

During *Staphylococcus aureus* infection, the rare human SNP R121W was identified to impair the interaction of MAL with MyD88, TLR-2 and TLR-4 (43). The effect of this SNP, initially predicted deleterious, resulted in increased compensatory adaptive immune responses and decreased invasive hematogenous infections in children (Figure 1C). This demonstrated that MAL not only affects innate immune responses but also adaptive immunity through not yet understood mechanisms.

## What Are the Interactions Between MAL and Pathogens?

To counteract immune response activation triggered by PAMPs detection by PRRs, several pathogenic bacteria express virulence factors, such as TIR domain-containing proteins, to perturb TIR-dependent interactions, which are essential in the initiation of innate immune responses (67–69). Salcedo's team demonstrated that *Pseudomonas aeruginosa* PA7 has a TIR domain-containing protein called PumA (Pseudomonas UBAP1 modulator A) conferring the ability to downmodulate innate immune responses (70). Indeed, PumA was translocated into host cells during infection to directly interact with MAL at the plasma membrane controlling TLR signaling. Similarly, *Brucella* produces a TIR domain-containing protein (TcpB/Btp1) to selectively target MAL and inhibit NF-κB activation, which is essential for intracellular *Brucella* survival and replication (Figure 1C) (71). Moreover, TcpB/Btp1 was also described to target CLIP170 enhancing MAL proteasome-mediated degradation (61). Furthermore, a number of viral proteins were described to interfere with innate immune signaling, highlighting the implication of the TLR pathway in antiviral immunity (72). Among them, the poxvirus protein A46 was identified to inhibit TLR-4 signaling by interfering physically with MAL (73, 74). It is of interest to investigate if other pathogens are also able to physically modulate MAL signaling to determine whether its deficiency could interfere with the progression of infection. To answer this question, bioinformatics might be useful to identify potential effectors that may interact with MAL.

## Is MAL Alone Responsible of Its Versatility?

Epistasis is a gene-gene interaction that influences a phenotype. As MAL interacts with numerous proteins, it is possible that genetic variations in these partners could modulate its signaling. Fulgione et al., investigated an epistatic interaction between MyD88 and MAL during *Helicobacter pylori* infection. A cohort study revealed that heterozygosity for S180L confer increased resistance to infection, which was found associated with a low level of IL-6, COX-2, TNF-α, and IL-1β production. Regardless of MAL, polymorphism in MyD88 alone did not influence the infection. However, in some combinations with MAL, MyD88 has an effect on the risk of infection. Together with MAL S180L, certain polymorphisms in MyD88 confer higher protection providing evidence of an epistatic interaction occurring between the two genes (44). The same polymorphic sites have also been documented to act epistatically against *Mtb* infection as well (29). These results showed that epistasis could play a key role in MAL versatility.

## Conclusion

Impairment of MAL expression in diseases result in some controversy. These discrepant findings about the differential effect of MAL during diseases could simply represent heterogeneity of association in different populations, which is well described for many immunogenetic polymorphisms (75). Frequencies of polymorphisms in *MAL* can vary among different races. This difference, along with gene-gene interactions, environmental and cultural factors, and variations in microbial strains make understanding the observed differences between ethnic groups even more complicated. Moreover, the size of the selected populations in different cohort studies can vary. This can impact the power of detection of small effects due to a rare mutation.

More investigations are still needed to characterize the contribution of MAL in each setting and to reach understanding of its impact on immune response to infection. In particular, the severity of the disease and its inflammatory status seem to have an essential impact. Deeper characterization of the local environments that are less favorable for the pathogen to survive may bring some cues.

In the emerging field of host-directed therapies to intracellular pathogens, TLRs and their adaptor proteins were proposed as putative targets for the treatment of inflammatory disorders and to overcome microbial resistance (76). For example, Gefitinib, Phycocianin and other peptides were recently studied for their inhibiting effect on MAL in the context of endotoxic injury, lung cancers and autoimmune diseases, respectively, showing promising results (77–80).

## Author Contributions

All authors participated in the concept, preparation, and writing of the manuscript.

## Conflict of Interest

The authors declare that the research was conducted in the absence of any commercial or financial relationships that could be construed as a potential conflict of interest.
